# Plasma miRNA expression profiles in rheumatoid arthritis associated interstitial lung disease

**DOI:** 10.1186/s12891-017-1389-4

**Published:** 2017-01-19

**Authors:** Shomi Oka, Hiroshi Furukawa, Kota Shimada, Atsushi Hashimoto, Akiko Komiya, Naoshi Fukui, Naoyuki Tsuchiya, Shigeto Tohma

**Affiliations:** 1 Molecular and Genetic Epidemiology Laboratory, Faculty of Medicine, University of Tsukuba, 1-1-1 Tennodai, Tsukuba, 305-8575 Japan; 2 Clinical Research Center for Allergy and Rheumatology, Sagamihara Hospital, National Hospital Organization, 18-1 Sakuradai, Minami-ku, Sagamihara 252-0392 Japan; 3 Department of Rheumatology, Sagamihara Hospital, National Hospital Organization, 18-1 Sakuradai, Minami-ku, Sagamihara 252-0392 Japan; 4 Department of Rheumatic Diseases, Tokyo Metropolitan Tama Medical Center, 2-8-29 Musashi-dai, Fuchu, 183-8524 Japan

**Keywords:** miRNA profile, Rheumatoid arthritis, Interstitial lung disease, Biological marker

## Abstract

**Background:**

Interstitial lung disease (ILD) is frequently associated with rheumatoid arthritis (RA), and is designated RA-associated ILD (RA-ILD). RA-ILD has a large impact on the prognosis of RA. Here, we investigated the micro RNAs (miRNAs) profiles to determine whether they may be useful for diagnosing RA-ILD.

**Methods:**

RNA was isolated from plasma samples and cDNA was synthesized. Real-time RT-PCR analysis was performed to evaluate 752 miRNA expression profiles in plasma pools from RA patients with or without RA-ILD. Sixteen selected miRNA levels were analyzed in individual plasmas from 64 RA patients with or without RA-ILD.

**Results:**

Expression levels of hsa-miR-214-5p (mean relative expression level ± standard deviation, 8.1 ± 28.2 in RA with ILD, 0.2 ± 0.9 in RA without ILD, *P* = 0.0156) and hsa-miR-7-5p (56.2 ± 260.4 in RA with ILD, 4.7 ± 11.8 in RA without ILD, *P* = 0.0362) were higher in RA patients with RA-ILD than in those without. The values of miRNA index (214, 7) generated from hsa-miR-214-5p and hsa-miR-7-5p for ILD were significantly elevated in RA patients with RA-ILD compared with those without (0.122 ± 0.332 in RA with ILD, 0.006 ± 0.013 in RA without ILD, *P* = 0.0010). The area under the curve value of the receiver operating characteristic curve for the miRNA index (214, 7) was 0.740.

**Conclusions:**

To the best of our knowledge, this is the first report of miRNA profiles in RA-ILD. The expression levels of hsa-miR-214-5p and hsa-miR-7-5p were increased in RA with ILD.

**Electronic supplementary material:**

The online version of this article (doi:10.1186/s12891-017-1389-4) contains supplementary material, which is available to authorized users.

## Background

Rheumatoid arthritis (RA) is a chronic systemic inflammatory autoimmune disease with bone and joint destruction. It is well known that interstitial lung disease (ILD) is frequently associated with RA [1]. Although nonspecific interstitial pneumonia (NSIP) pattern is predominantly observed in collagen disease patients with ILD, usual interstitial pneumonia (UIP) pattern is often observed in RA-associated ILD (RA-ILD) patients [2]. The prognosis of idiopathic interstitial pneumonia patients with UIP pattern was reported to be worse than those with NSIP [3]. The presence of ILD influences the prognosis of RA [4, 5]. Krebs von den lungen-6 (KL-6) and surfactant protein-D (SP-D) have been used as biomarkers for ILD, but have low sensitivity for the detection of RA-ILD [6, 7]. Thus, better biomarkers for the early screening of RA-ILD are eagerly expected.

Micro RNAs (miRNAs) are small non-coding RNAs with approximate 22 nucleotide length and are stably detected in plasma or serum. It is widely known that miRNAs modulate the expression of protein-coding genes at the post-transcription level and play important roles in cell activation, proliferation, differentiation, or death. Dysregulation of miRNAs in the circulation was detected in the patients with cancer or other diseases and circulating miRNAs could be potential biomarkers for various diseases [8–10]. Some miRNAs in the circulation were also dysregulated in inflammatory diseases, such as RA [11, 12], inflammatory bowel diseases [13], or idiopathic pulmonary fibrosis (IPF) [14, 15], though the impact of the circulating miRNA in inflammatory diseases does not reach to that in cancer. The expression levels of hsa-miR-132, hsa-miR-24, and hsa-miR-125a-5p were altered in plasma from RA patients [12, 16]. The expression levels of hsa-miR-21 were increased in sera from IPF patients [14, 15, 17]. However, few studies have focused on circulating miRNA profiles of RA-ILD. The present study investigated circulating miRNA profiles of RA-ILD to determine whether they may be useful for diagnosing RA-ILD.

## Methods

### Patients

Sixty four Japanese patients with RA were recruited at Sagamihara Hospital. ILD was diagnosed from computed tomography (CT) findings by two physicians specializing in RA-ILD. RA patients were categorized from A to Z, based on the Sagamihara Criteria [1]. RA cases in categories A to D were RA with ILD [ILD(+)RA] and those in G and H were RA without ILD [ILD(−)RA]. This study included RA cases in categories A [Findings consistent with ILD were observed in high resolution CT (HRCT) images (length of shorter diameter of the lesion was ≥2 cm in a transverse section)] or H [HRCT images were normal] [1]. RA patients with ILD were further diagnosed with one of the two patterns of UIP or NSIP, based on the predominant CT findings: UIP, irregular linear opacities and honeycombing; NSIP, bilateral ground-glass attenuation patterns predominantly in subpleural and basal regions [2, 18, 19]. Plasma samples from the 64 RA patients with or without ILD were collected and these individual plasma samples were analyzed for miRNA expression profiles. Blood samples were taken in tubes with ethylenediaminetetraacetic acid dipotassium salt (Terumo, Tokyo, Japan) and kept in room temperature before separation. Plasma was separated by centrifugation at 1500 × g for 10 min, and then stored at −80 °C until analysis. All patients fulfilled the American College of Rheumatology criteria for RA [20]. This study was reviewed and approved by Sagamihara Hospital Research Ethics Committee and University of Tsukuba Research Ethics Committee. Written informed consent was obtained from all study participants. This study was conducted in accordance with the principles expressed in the Declaration of Helsinki.

### MiRNA analysis

RNA was isolated from 200 μl plasma samples using miRCURY RNA Isolation kit Biofluids (Exiqon, Vedbaek, Denmark) and complementary DNA (cDNA) was synthesized with miRCURY LNA Universal cDNA Synthesis kit II (Exiqon). Real-time RT-PCR analysis was performed to evaluate miRNA expression in the plasma pool from 17 RA patients with RA-ILD or the pool from 17 RA patients without ILD, using Human miRNome microRNA PCR Panel I + II (Exiqon), ExiLENT SYBR Green master mix (Exiqon), and LightCycler 480 System II (Roche, Basel, Switzerland). Thermal cycling conditions consisted of initial denaturation at 95 °C for 10 min, followed by 45 cycles of 95 °C for 10 s followed by 60 °C for 1 min. Interplate calibration was conducted by the subtraction of the average Cт values of UniSP3 probe of the plate. The levels of miRNA among plasma samples were normalized to the average expression of five circulating miRNAs; hsa-miR-425-5p, hsa-miR-423-5p, hsa-miR-103a-3p, hsa-miR-191-5p, hsa-miR-93-5p. The amount of miRNA was quantified using comparative Cт method. The two pooled plasma of ILD(+)RA or ILD(−)RA were screened for the miRNA profiling. The data obtained from the microRNA PCR panels were deposited in Gene Expression Omnibus of National Center for Biotechnology Information and are accessible by accession number GSE88899. The PCR panel contains 752 probes for human miRNAs. Potential miRNA markers were selected for real-time RT-PCR analysis of miRNAs in individual patient plasma, based on the PCR panel data; eight miRNAs with higher absolute _⊿⊿_Cт values (The _⊿⊿_Cт values of these eight miRNAs were higher than 3.12) were selected when the samples with undefined Cт values of wells for detection of gene of interest were eliminated for the selection. Other eight miRNAs with higher absolute_⊿⊿_Cт values (The _⊿⊿_Cт values of these eight miRNAs were higher than 5.10) were selected when the undefined C_T_ values were substituted by 40. Thus, sixteen miRNAs were selected for the individual analysis.

Pick-&-Mix microRNA PCR Panel (Exiqon), ExiLENT SYBR Green master mix (Exiqon), and Applied Biosystems 7500 Fast Real-Time PCR System (Thermo Fisher Scientific, Waltham, MA, USA) were employed for the detection of miRNAs in each individual plasma sample from 64 RA patients with or without RA-ILD that include the above-mentioned 34 RA patients in the screening. The same thermal cycling condition was used. The expression levels of miRNAs in the samples with undefined Cт values were define to be 0. The levels of miRNA among plasma samples were normalized to the average expression of three circulating miRNAs; hsa-miR-103a-3p, hsa-miR-191-5p, hsa-miR-93-5p. Relative expression levels of miRNAs in plasma were calculated with comparative Cт method.

### Statistical analysis

Differences in patient characteristics were analyzed by Mann-Whitney’s U test or Fisher’s exact test using 2X2 contingency tables. Mann-Whitney’s U test was conducted for the comparison of miRNA expression levels. Multiple linear regression analysis was performed to develop miRNA index for ILD in RA from miRNA levels. Correction for multiple testing was performed by calculating false discovery rate Q-value [21]. Target genes for the miRNAs were predicted using target prediction algorithm at miRDB (http://mirdb.org) [22].

## Results

### Clinical features of the RA patients

Characteristics of the RA patients are shown in Table 1. Mean age, percentage of males, KL-6, SP-D, and disease activity score 28 (DAS28) in ILD(+)RA were higher than in ILD(−)RA. The percentage of administration of biological disease-modifying anti-rheumatic drugs (DMARDs) in ILD(+)RA were lower than in ILD(−)RA. Mean age, percentage of males, KL-6, and SP-D in RA with ILD of UIP pattern [UIP(+)RA] were higher than in ILD(−)RA. The percentage of administration of biological DMARDs in UIP(+)RA were lower than in ILD(−)RA. Mean age, disease duration, KL-6, SP-D, DAS28, and DAS28CRP in RA with ILD of NSIP pattern [NSIP(+)RA] were higher than in ILD(−)RA.

**Table 1:** Characteristics of the RA patients

						ILD(+)RA	UIP(+)RA	NSIP(+)RA
		ILD(+)RA	UIP(+)RA	NSIP(+)RA	ILD(−)RA	*P*	*P*	*P*
Number		32	18	14	32			
Age	year	70.2 (7.4)	70.6 (8.0)	69.6 (6.8)	57.7 (13.8)	0.0002	0.0013	0.0043
male number	n (%)	11 (34.4)	9 (50.0)	2 (14.3)	2 (6.3)	*0.0109	*0.0007	*0.5745
Disease duration	year	15.5 (12.6)	12.8 (12.2)	18.9 (12.7)	10.6 (7.4)	0.1830	0.8953	0.0116
Steinbrocker stage III and IV	n (%)	18 (56.3)	9 (50.0)	9 (64.3)	21 (65.6)	*0.6088	*0.3701	*1.0000
Smoker or past smoker	n (%)	10 (33.3)	7 (38.9)	3 (25.0)	10 (32.3)	*1.0000	*0.7581	*0.7272
Rheumatoid factor positive	n (%)	30 (93.8)	17 (94.4)	13 (92.9)	27 (84.4)	*0.4258	*0.3991	*0.6506
Anti-citrullinated peptide antibody positive	n (%)	31 (96.9)	18 (100.0)	13 (92.9)	31 (96.9)	*1.0000	*1.0000	*0.5208
KL-6	U/ml	1096.4 (853.7)	1086.2 (828.4)	1108.8 (914.9)	315.3 (257.9)	2.44 × 10^−7^	2.97 × 10^−6^	0.0001
SP-D	ng/ml	194.1 (244.7)	177.3 (192.3)	214.5 (303.0)	46.0 (37.5)	2.48 × 10^−5^	0.0008	0.0001
DAS28		4.0 (1.2)	3.7 (1.3)	4.3 (0.9)	3.1 (1.2)	0.0040	0.0793	0.0021
DAS28-CRP		3.0 (1.3)	2.6 (1.4)	3.4 (1.0)	2.4 (1.0)	0.0500	0.5855	0.0035
NSAIDs administration	n (%)	13 (41.9)	7 (38.9)	6 (46.2)	15 (48.4)	*0.7989	*0.5647	*1.0000
Corticosteroid administration	n (%)	26 (83.9)	14 (77.8)	12 (92.3)	25 (80.6)	*1.0000	*1.0000	*0.6542
DMARDs administration	n (%)	24 (77.4)	14 (77.8)	10 (76.9)	30 (96.8)	*0.0529	*0.0542	*0.0706
sDMARDs administration	n (%)	19 (61.3)	11 (61.1)	8 (61.5)	19 (61.3)	*1.0000	*1.0000	*1.0000
bDMARDs administration	n (%)	10 (32.3)	5 (27.8)	5 (38.5)	22 (71.0)	*0.0048	*0.0066	*0.0874

Average values or numbers of each group are shown. Standard deviations or percentages are shown in parenthesis. Differences were tested by Mann-Whitney’s U test or Fisher’s exact test using 2 × 2 contingency tables

*RA* rheumatoid arthritis, *ILD* interstitial lung disease, *UIP* Usual interstitial pneumonia, *NSIP* Nonspecific interstitial pneumonia, *ILD(+)RA* RA with ILD, *UIP(+)RA* RA with ILD of UIP pattern, *NSIP(+)RA* RA with ILD of NSIP pattern, *ILD(−)RA* RA without ILD, *KL-6* krebs von den lungen-6, *SP-D* Surfactant protein-D, *DAS28* disease activity score 28, *NSAIDs* non-steroidal anti- inflammatory drug, *DMARD* disease-modifying anti-rheumatic drug, *sDMARD* synthetic DMARD, *bDMARD* biological DMARD

*Fisher’s exact test was employed

### MiRNA expression profiles of RA patients with or without ILD

Pooled plasma miRNA profiles were compared between ILD(+)RA and ILD(−)RA groups (Additional file 1: Table S1). Of 752 tested miRNA, 530 miRNAs were up-regulated and 217 miRNAs were down-regulated in ILD(+)RA. Sixteen miRNAs with higher absolute _⊿⊿_Cт values were selected for further analysis on the individual plasmas, as described in Materials and methods (indicated in red, Additional file 1: Table S1).

**Fig. 1:**
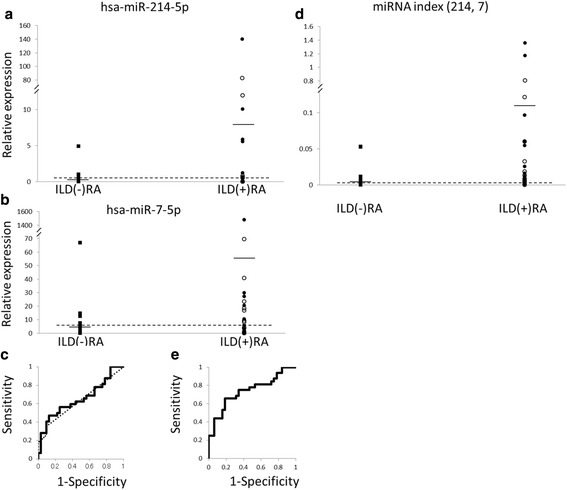
Evaluation of the miRNA expression levels, as a marker for interstitial lung disease (ILD) in rheumatoid arthritis (RA) patients. **a** Distribution of the hsa-miR-214-5p. The *filled square*, *filled circle*, and *empty circle* represent RA without ILD, RA with usual interstitial pneumonia, and RA with nonspecific interstitial pneumonia, respectively. *Horizontal bars* denote means. The *horizontal dotted line* represents an optimized cut-off level (hsa-miR-214-5p = 0.429, with specificity and sensitivity of 0.906 and 0.375, respectively). ILD(+)RA: RA with ILD, ILD(−)RA: RA without ILD. **b** Distribution of the hsa-miR-7-5p. The *horizontal dotted line* represents an optimized cut-off level (hsa-miR-7-5p = 5.686, with specificity and sensitivity of 0.875 and 0.469, respectively). **c** The receiver operating characteristic (ROC) curve using the hsa-miR-214-5p (*dotted line*) and hsa-miR-7-5p (*solid line*) as markers for ILD in RA. The area under the curve (AUC) value of the ROC curve for hsa-miR-214-5p is 0.634 and that for hsa-miR-7-5p is 0.652. **d** Distribution of miRNA index (214, 7) generated from hsa-miR-214-5p and hsa-miR-7-5p for ILD (=0.0095 X hsa-miR-214-5p + 0.0008 X hsa-miR-7-5p). The *horizontal dotted line* represents an optimized cut-off level (miRNA index (214, 7) = 0.004, with specificity and sensitivity of 0.813 and 0.656, respectively). **e** The ROC curve using miRNA index (214, 7) as a marker for ILD in RA. The AUC value of the ROC curve is 0.740

Sixteen miRNAs as potential biomarkers (hsa-miR-29c-3p, hsa-miR-154-5p, hsa-miR-543, hsa-miR-214-5p, hsa-miR-382-3p, hsa-let-7g-3p, hsa-miR-9-5p, hsa-miR-370-3p, hsa-miR-221-5p, hsa-miR-483-5p, hsa-miR-7-5p, hsa-miR-376b-3p, hsa-miR-487b-3p, hsa-let-7f-1-3p, hsa-miR-500a-5p, hsa-miR-582-5p) were selected for further analysis of miRNAs in individual patient plasma. The 16 miRNAs were also compared between the ILD(+)RA and ILD(−)RA using the real-time RT-PCR method (Table 2 and Additional file 2: Figure S1). The results show a tendency for the expression levels of hsa-miR-214-5p and hsa-miR-7-5p to be increased in the ILD(+)RA group (Fig. 1a and b). Although, male percentage is higher in the ILD(+)RA group, the expression levels of 16 selected miRNAs were not different between male and female in the ILD(+)RA group (Additional file 3: Table S2). The effects of the age on the expression levels of some miRNAs were detected (Additional file 4: Table S3). The effects of the DAS28 on the expression levels of miRNAs could not be detected in the present study (Additional file 5: Table S4). The effects of the biological DMARDs on the expression levels of miRNA were not found (Additional file 6: Table S5). For hsa-miR-214-5p, 135 target genes were predicted and include *SMAD4*, coding a protein involved in signal transduction of the transforming growth factor β [23]. For hsa-miR-7-5p, 434 target genes were predicted and include *BLOC1S4*. The mutation of *Bloc1s4* causes a model of Hermansky-Pudlak syndrome [24].

**Table 2:** miRNA profiles of the RA patients with or without ILD

					ILD(+)RA		UIP(+)RA		NSIP(+)RA	
miRNA	ILD(+)RA	UIP(+)RA	NSIP(+)RA	ILD(−)RA	*P*	*FDR Q*	*P*	*FDR Q*	*P*	*FDR Q*
hsa-miR-29c-3p	28.7 (92.4)	45.9 (121.6)	6.7 (10.4)	2.9 (2.5)	0.1772	0.6025	0.0499	0.1698	0.9334	0.9334
hsa-miR-154-5p	17.9 (59.1)	24.6 (76.8)	9.3 (21.3)	2.0 (2.8)	0.9238	0.9815	0.5331	0.7294	0.3604	0.7416
hsa-miR-543	11.0 (35.9)	14.8 (46.6)	6.1 (13.9)	1.4 (1.4)	0.4386	0.6213	0.8633	0.9173	0.1145	0.6099
hsa-miR-214-5p	8.1 (28.2)	13.6 (37.0)	1.0 (3.2)	0.2 (0.9)	0.0156	0.1322	0.0076	0.0643	0.1697	0.6099
hsa-miR-382-3p	10.9 (41.5)	14.8 (52.9)	5.8 (19.9)	0.9 (1.7)	0.4279	0.6213	0.9473	0.9473	0.1794	0.6099
hsa-let-7g-3p	29.1 (107.3)	50.3 (141.1)	1.9 (3.7)	3.3 (5.3)	0.6098	0.7974	0.5578	0.7294	0.1095	0.6099
hsa-miR-9-5p	5.8 (18.5)	9.1 (24.2)	1.5 (4.2)	0.9 (2.6)	0.8510	0.9815	0.7724	0.8753	0.4947	0.7416
hsa-miR-370-3p	12.2 (57.4)	18.2 (76.3)	4.5 (11.1)	0.3 (0.4)	0.8766	0.9815	0.3648	0.6202	0.4734	0.7416
hsa-miR-221-5p	17.1 (53.1)	26.6 (68.7)	5.0 (16.6)	0.7 (1.2)	0.3227	0.6213	0.0461	0.1698	0.5235	0.7416
hsa-miR-483-5p	80.7 (331.8)	130.5 (440.8)	16.6 (24.4)	14.3 (33.9)	0.2827	0.6213	0.2330	0.4951	0.6161	0.7888
hsa-miR-7-5p	56.2 (260.4)	91.1 (347.0)	11.5 (19.9)	4.7 (11.8)	0.0362	0.2049	0.0275	0.1559	0.2616	0.6571
hsa-miR-376b-3p	14.6 (48.8)	19.0 (63.4)	9.0 (19.3)	1.1 (1.4)	0.3718	0.6213	0.2621	0.4951	0.7915	0.8751
hsa-miR-487b-3p	12.7 (56.4)	19.1 (75.0)	4.4 (10.2)	0.5 (0.7)	0.2437	0.6213	0.1181	0.2868	0.8237	0.8751
hsa-let-7f-1-3p	24.9 (114.7)	39.8 (153.0)	5.7 (9.4)	2.6 (2.8)	0.9893	0.9893	0.6856	0.8326	0.6496	0.7888
hsa-miR-500a-5p	55.8 (230.6)	94.7 (305.3)	5.7 (12.3)	1.7 (4.6)	0.3744	0.6213	0.4566	0.7056	0.4823	0.7416
hsa-miR-582-5p	144.8 (288.5)	208.4 (360.8)	63.0 (124.4)	31.5 (60.5)	0.0693	0.2946	0.0705	0.1998	0.2706	0.6571
miRNA Index (214, 7)	0.122 (0.332)	0.202 (0.430)	0.019 (0.034)	0.006 (0.013)	0.0010	0.0162	0.0005	0.0093	0.0732	0.6099

Average values of each group are shown. Standard deviations are shown in parenthesis. Differences were tested by Mann-Whitney’s U test. To correct for multiple testing, the false discovery rate Q-value was calculated

*RA* rheumatoid arthritis, *ILD* interstitial lung disease, *UIP* Usual interstitial pneumonia, *NSIP* Nonspecific interstitial pneumonia, *ILD(+)RA* RA with ILD, *UIP(+)RA* RA with ILD of UIP pattern, *NSIP(+)RA* RA with ILD of NSIP pattern, *ILD(−)RA* RA without ILD

The area under the curve (AUC) value of the receiver operating characteristic (ROC) curve for hsa-miR-214-5p was 0.634 and that for hsa-miR-7-5p was 0.652 (Fig. 1c). Specificities and sensitivities of these miRNAs were estimated from the ROC curve conditional on the highest Youden’s index. The optimized cut-off level of hsa-miR-214-5p was 0.429 with the sensitivity (0.375) and the specificity (0.906). The optimized cut-off level of hsa-miR-7-5p was 5.686 with the sensitivity (0.469) and the specificity (0.875).

An miRNA index (214, 7) was generated from the expression levels of hsa-miR-214-5p and hsa-miR-7-5p and evaluated as a marker for ILD(+)RA; miRNA index (214, 7) = 0.0095 X hsa-miR-214-5p + 0.0008 X hsa-miR-7-5p. The miRNA index (214, 7) was higher in the ILD(+)RA group (Fig. 1d, Table 2, *P* =0.0010, *Q* = 0.0162, mean ± SD = 0.122 ± 0.332 in ILD(+)RA, 0.006 ± 0.013 in ILD(−)RA). The AUC value of the ROC curve for the miRNA index (214, 7) was 0.740 (Fig. 1e). Specificities and sensitivities of miRNA index (214, 7) were estimated from the ROC curve conditional on the highest Youden’s index. The optimized cut-off level was 0.004 with the sensitivity (0.656) and specificity (0.813). Thus, the plasma miRNA profiles were different between ILD(+)RA and ILD(−)RA groups.

### MiRNA expression profiles of RA patients with UIP or NSIP patterns

The expression levels of the 16 miRNAs were compared between the UIP(+)RA and ILD(−)RA. A tendency for the expression levels of hsa-miR-214-5p and hsa-miR-7-5p to be increased in the UIP(+)RA group was also observed. The values of miRNA index (214, 7) in the UIP(+)RA group was higher (*P* = 0.0005, *Q* = 0.0093, mean ± SD = 0.202 ± 0.430 in UIP(+)RA). The expression pattern of the 16 miRNAs were also compared between the NSIP(+)RA and ILD(−)RA. No association was observed in the NSIP(+)RA group. Thus, miRNA profiles were also altered in UIP(+)RA groups.

## Discussion

It was reported that plasma miRNA profiles are altered not only in cancerous patients [8–10], but also in patients with inflammatory diseases [11–15]. However, few studies have focused on miRNA profiling in RA-ILD, though these could be diagnostic markers overcoming the existing markers with low sensitivity [6, 7]. We have tried to discriminate RA-ILD, one of the potentially life-threatening extra-articular manifestations of RA, using plasma miRNA profiles. To the best of our knowledge, this is the first report of plasma miRNA profiles in RA-ILD. We found that hsa-miR-214-5p and hsa-miR-7-5p were increased in the ILD(+)RA group (Fig. 1a and b), though the superiority of these miRNA profiles was not observed compared with KL-6 (AUC = 0.86, cut-off level = 331.5, sensitivity =0.769, and specificity =0.842) [7].

It was already well known that cancer cells produce circulating extracellular miRNAs in plasma samples from cancerous patients. Inflammatory cells or tissues may also produce circulating extracellular miRNAs in plasma samples from patients with inflammatory diseases. Thus, hsa-miR-214-5p and hsa-miR-7-5p might be preferentially produced by inflammatory cells or tissues. Though the expression levels of hsa-miR-21 were increased in sera from IPF patients [14, 15, 17], this increase was not observed in the pooled sera from the ILD(+)RA group (Additional file 1: Table S1). This discrepancy could be explained by the difference between the pathogenesis of IPF that includes only UIP and that of RA-ILD that includes both UIP and NSIP.

Since miRNAs modulate the expression of protein-coding genes at the post-transcription level, many studies on the expression and the function of miRNAs were reported. It was reported that the expression levels of hsa-miR-214-5p were increased in B cell lymphoma with better prognosis [25] and liver cirrhosis [26], but decreased in hepatocellular carcinoma [27]. The expression of hsa-miR-7-5p inhibits melanoma cell migration [28], and the decreased expression levels of hsa-miR-7-5p in follicular thyroid cancer were thought to be a reliable marker [29]. The expression levels of hsa-miR-7-5p were increased in dental pulp stem cells [30]. The altered expression levels of hsa-miR-7-5p were also reported in breast cancer and glioblastoma [31–33]. Thus, the altered expression patterns and the function of hsa-miR-214-5p and hsa-miR-7-5p have been reported. The target genes of the two miRNAs were predicted. Although many genes were predicted, some predicted genes, including *SMAD4* and *BLOC1S4*, are known to be involved in the pathogenesis of ILD. However, further functional studies on these miRNAs would be expected to provide better understanding of the roles of these miRNA for the pathogenesis of RA-ILD.

Because of the limited sample size, the validation of miRNA profiles should be performed in future independent work. For the practical applications of miRNA biomarkers for RA-ILD, the expression patterns of all miRNAs should be comprehensively investigated. Therefore, further large-scale miRNA profiling using next generation sequencer could be expected.

## Conclusions

This is the first report of plasma miRNA profiles of RA-ILD. The expression levels of hsa-miR-214-5p and hsa-miR-7-5p were increased in the ILD(+)RA group and they could be potential biomarkers for ILD in RA.

## Additional files

Additional file 1: Table S1. miRNA expression ratios in plasmas from the RA patients with or without ILD. *Undifined Cтvalues were substituted by 40. Selected miRNAs for further analyses were indicated in red (DOCX 82 kb)
Additional file 2: Figure S1. Distribution of the miRNA, Krebs von den lungen-6 (KL-6), surfactant protein-D (SP-D), ILDIndex levels, as markers for interstitial lung disease (ILD) in rheumatoid arthritis (RA) patients. The filled square, filled circle, and empty circle represent ILD(−)RA, RA with usual interstitial pneumonia, and RA with nonspecific interstitial pneumonia, respectively. ILD(+)RA: RA with ILD, ILD(−)RA: RA without ILD. (TIFF 687 kb)
Additional file 3: Table S2. miRNA profiles of the RA patients with ILD. Standard deviations are shown in parenthesis. Difference were tested between male and female by Mann-Whitney’s U test. RA: rheumatoid arthritis, ILD(+)RA: ILD positive RA. Average values of each group are shown. (DOCX 15 kb)
Additional file 4: Table S3. miRNA profiles of the RA patients with ILD. Average values of each group are shown. Standard deviations are shown in parenthesis. Difference were tested between age < 70 and age ≧ 70 by Mann-Whitney’s U test. RA: rheumatoid arthritis, ILD(+)RA: ILD positive RA. (DOCX 15 kb)
Additional file 5: Table S4. miRNA profiles of the RA patients with ILD. Average values of each group are shown. Standard deviations are shown in parenthesis. Difference were tested between DAS28 < 4.0 and DAS28 ≧ 4.0 by Mann-Whitney’s U test. RA: rheumatoid arthritis, ILD(+)RA: ILD positive RA. (DOCX 15 kb)
Additional file 6: Table S5. miRNA profiles of the RA patients with ILD. Average values of each group are shown. Standard deviations are shown in parenthesis. Difference were tested between bDMARDs(+) and bDMARDs(−) by Mann-Whitney’s U test. RA: rheumatoid arthritis, ILD(+)RA: ILD positive RA. (DOCX 15 kb)

## Abbreviations

AUC: Area under the curve
cDNA: Complementary DNA
CT: Computed tomography
HRCT: High resolution CT
ILD: Interstitial lung disease
ILD(+)RA: RA with ILD
ILD(−)RA: RA without ILD
IPF: Idiopathic pulmonary fibrosis
KL-6: Krebs von den lungen-6
miRNA: Micro RNA
NSIP: Nonspecific interstitial pneumonia
NSIP(+)RA: RA with ILD of NSIP pattern
RA: Rheumatoid arthritis
RA-ILD: RA-associated ILD
ROC: Receiver operating characteristic
SP-D: Surfactant protein-D
UIP: Usual interstitial pneumonia
UIP(+)RA: RA with ILD of UIP pattern
