# Supplementary material for: Plasma miRNA expression profiles in rheumatoid arthritis associated interstitial lung disease
Source: BMC Musculoskelet Disord. 2017 Jan 19;18:21. doi: 10.1186/s12891-017-1389-4 (PMC5244611; doi:10.1186/s12891-017-1389-4)
Supplement: Additional file 1: Table S1. — miRNA expression ratios in plasmas from the RA patients with or without ILD. *Undifined Cтvalues were substituted by 40. Selected miRNAs for further analyses were indicated in red (DOCX 82 kb) [file 12891_2017_1389_MOESM1_ESM.docx]

| Supplementary Table 1. miRNA expression ratios in plasmas from the RA patients with or without ILD. | | |  |  |  |
| --- | --- | --- | --- | --- | --- |
| miRNA name | Expression ratio (log_2_) | |  |  |  |
| hsa-miR-29c-3p | 12.0920 | * |  |  |  |
| hsa-miR-154-5p | -7.3080 | * |  |  |  |
| hsa-miR-543 | -6.9113 | * |  |  |  |
| hsa-miR-214-5p | 6.4687 | * |  |  |  |
| hsa-miR-382-3p | 5.6387 | * |  |  |  |
| hsa-let-7g-3p | 5.5787 | * |  |  |  |
| hsa-miR-9-5p | 5.2720 | * |  |  |  |
| hsa-miR-370-3p | 5.1320 | * |  |  |  |
| hsa-miR-499a-5p | 5.0920 | * |  |  |  |
| hsa-miR-99a-3p | 5.0920 | * |  |  |  |
| hsa-miR-212-3p | 5.0720 | * |  |  |  |
| hsa-miR-203a | 5.0720 | * |  |  |  |
| hsa-miR-618 | 4.8587 | * |  |  |  |
| hsa-miR-221-5p | 4.6887 |  |  |  |  |
| hsa-miR-665 | 4.6120 | * |  |  |  |
| hsa-miR-31-5p | 4.5820 | * |  |  |  |
| hsa-miR-34b-3p | 4.5720 | * |  |  |  |
| hsa-miR-493-5p | -4.5413 | * |  |  |  |
| hsa-miR-129-1-3p | 4.5087 | * |  |  |  |
| hsa-miR-873-5p | 4.4220 | * |  |  |  |
| hsa-miR-301b | -4.3880 | * |  |  |  |
| hsa-miR-411-5p | -4.2380 | * |  |  |  |
| hsa-miR-190b | 4.2187 | * |  |  |  |
| hsa-miR-548h-5p | 4.1787 | * |  |  |  |
| hsa-miR-1203 | 4.1587 | * |  |  |  |
| hsa-miR-509-3p | -3.9580 | * |  |  |  |
| hsa-miR-483-5p | 3.9387 |  |  |  |  |
| hsa-miR-7-5p | 3.9320 |  |  |  |  |
| hsa-miR-122-3p | -3.9313 | * |  |  |  |
| hsa-miR-1255b-5p | 3.8787 | * |  |  |  |
| hsa-miR-196b-5p | -3.8580 | * |  |  |  |
| hsa-miR-105-3p | 3.6887 | * |  |  |  |
| hsa-miR-381-3p | -3.6780 | * |  |  |  |
| hsa-miR-604 | 3.6587 | * |  |  |  |
| hsa-miR-181a-2-3p | -3.6413 | * |  |  |  |
| hsa-miR-224-3p | -3.6413 | * |  |  |  |
| hsa-miR-493-3p | -3.6280 | * |  |  |  |
| hsa-miR-23b-5p | -3.6113 | * |  |  |  |
| hsa-miR-483-3p | -3.5780 | * |  |  |  |
| hsa-miR-376b-3p | -3.5580 |  |  |  |  |
| hsa-miR-196a-5p | 3.5120 | * |  |  |  |
| hsa-miR-196b-3p | -3.4213 | * |  |  |  |
| hsa-miR-377-5p | -3.4113 | * |  |  |  |
| hsa-miR-200b-3p | 3.3620 | * |  |  |  |
| hsa-miR-29a-5p | -3.3113 | * |  |  |  |
| hsa-miR-570-3p | -3.3080 | * |  |  |  |
| hsa-miR-379-3p | -3.2813 | * |  |  |  |
| hsa-miR-331-5p | 3.2687 | * |  |  |  |
| hsa-miR-487b-3p | -3.2680 |  |  |  |  |
| hsa-miR-187-3p | -3.2380 | * |  |  |  |
| hsa-let-7f-1-3p | 3.2087 |  |  |  |  |
| hsa-miR-501-5p | -3.1880 | * |  |  |  |
| hsa-miR-500a-5p | 3.1720 |  |  |  |  |
| hsa-miR-200c-3p | -3.1680 | * |  |  |  |
| hsa-miR-299-5p | -3.1480 | * |  |  |  |
| hsa-miR-582-5p | 3.1420 |  |  |  |  |
| hsa-miR-376a-3p | -3.1180 |  |  |  |  |
| hsa-miR-452-5p | -3.0880 | * |  |  |  |
| hsa-miR-24-1-5p | 3.0687 | * |  |  |  |
| hsa-miR-200a-3p | -3.0680 | * |  |  |  |
| hsa-miR-96-5p | 3.0520 |  |  |  |  |
| hsa-miR-136-3p | -3.0413 |  |  |  |  |
| hsa-miR-134 | -2.9980 |  |  |  |  |
| hsa-miR-190a | 2.9220 | * |  |  |  |
| hsa-miR-1271-5p | -2.7913 | * |  |  |  |
| hsa-miR-1249 | -2.7913 |  |  |  |  |
| hsa-miR-576-3p | -2.7880 | * |  |  |  |
| hsa-miR-495-3p | -2.7480 |  |  |  |  |
| hsa-miR-95 | -2.7280 | * |  |  |  |
| hsa-miR-708-3p | 2.7087 | * |  |  |  |
| hsa-miR-136-5p | -2.7080 |  |  |  |  |
| hsa-miR-369-5p | -2.6980 | * |  |  |  |
| hsa-miR-585 | -2.6713 | * |  |  |  |
| hsa-miR-941 | -2.6513 | * |  |  |  |
| hsa-miR-760 | -2.6380 | * |  |  |  |
| hsa-miR-16-1-3p | 2.6220 |  |  |  |  |
| hsa-miR-487a | -2.6113 | * |  |  |  |
| hsa-miR-377-3p | -2.6080 |  |  |  |  |
| hsa-miR-654-3p | -2.5913 |  |  |  |  |
| hsa-let-7a-3p | -2.5713 | * |  |  |  |
| hsa-miR-548k | -2.5613 | * |  |  |  |
| hsa-miR-101-5p | 2.5587 |  |  |  |  |
| hsa-miR-19a-5p | -2.5513 | * |  |  |  |
| hsa-miR-654-5p | -2.5480 | * |  |  |  |
| hsa-miR-323a-3p | -2.4480 |  |  |  |  |
| hsa-miR-193a-5p | 2.4120 |  |  |  |  |
| hsa-miR-153 | -2.3980 |  |  |  |  |
| hsa-miR-629-5p | 2.3720 |  |  |  |  |
| hsa-miR-551b-3p | -2.3580 |  |  |  |  |
| hsa-miR-1972 | 2.2187 |  |  |  |  |
| hsa-miR-454-5p | -2.2013 | * |  |  |  |
| hsa-miR-605 | 2.1987 |  |  |  |  |
| hsa-miR-1185-5p | -2.1813 | * |  |  |  |
| hsa-miR-125b-2-3p | -2.1713 | * |  |  |  |
| hsa-miR-181a-3p | -2.1680 |  |  |  |  |
| hsa-miR-363-3p | 2.1520 |  |  |  |  |
| hsa-miR-548d-5p | -2.1413 | * |  |  |  |
| hsa-miR-380-3p | -2.1313 | * |  |  |  |
| hsa-miR-675-3p | -2.1213 | * |  |  |  |
| hsa-miR-135a-5p | -2.1080 | * |  |  |  |
| hsa-miR-362-3p | 2.0987 |  |  |  |  |
| hsa-miR-374b-5p | 2.0920 |  |  |  |  |
| hsa-miR-1245a | 2.0887 |  |  |  |  |
| hsa-miR-579 | 2.0587 |  |  |  |  |
| hsa-miR-205-5p | 2.0520 |  |  |  |  |
| hsa-miR-32-3p | -2.0513 |  |  |  |  |
| hsa-miR-24-2-5p | 2.0287 |  |  |  |  |
| hsa-miR-409-5p | -2.0213 | * |  |  |  |
| hsa-miR-641 | -2.0013 | * |  |  |  |
| hsa-miR-181c-5p | 1.9820 |  |  |  |  |
| hsa-miR-431-5p | -1.9580 |  |  |  |  |
| hsa-miR-342-5p | 1.9387 |  |  |  |  |
| hsa-miR-539-5p | -1.9280 | * |  |  |  |
| hsa-miR-449a | -1.9280 | * |  |  |  |
| hsa-miR-592 | -1.9213 | * |  |  |  |
| hsa-miR-25-5p | -1.9213 | * |  |  |  |
| hsa-miR-556-3p | 1.9187 |  |  |  |  |
| hsa-miR-432-5p | -1.9080 |  |  |  |  |
| hsa-miR-376c-3p | -1.8980 |  |  |  |  |
| hsa-miR-502-3p | 1.8787 |  |  |  |  |
| hsa-miR-410 | -1.8680 |  |  |  |  |
| hsa-miR-182-5p | 1.8320 |  |  |  |  |
| hsa-miR-127-5p | -1.8180 | * |  |  |  |
| hsa-miR-29c-5p | -1.8013 |  |  |  |  |
| hsa-miR-632 | 1.7687 |  |  |  |  |
| hsa-miR-18a-3p | -1.7380 |  |  |  |  |
| hsa-miR-30c-1-3p | -1.7313 | * |  |  |  |
| hsa-miR-373-3p | -1.7080 | * |  |  |  |
| hsa-miR-589-3p | -1.7013 | * |  |  |  |
| hsa-miR-219-5p | -1.6780 | * |  |  |  |
| hsa-miR-9-3p | 1.6720 |  |  |  |  |
| hsa-miR-1179 | -1.6713 | * |  |  |  |
| hsa-miR-27a-5p | 1.6687 |  |  |  |  |
| hsa-miR-340-3p | -1.6513 | * |  |  |  |
| hsa-miR-424-5p | 1.6420 |  |  |  |  |
| hsa-miR-30a-3p | 1.6087 |  |  |  |  |
| hsa-miR-26a-1-3p | -1.5713 |  |  |  |  |
| hsa-miR-934 | -1.5380 | * |  |  |  |
| hsa-miR-140-3p | 1.5320 |  |  |  |  |
| hsa-let-7i-3p | 1.5187 |  |  |  |  |
| hsa-miR-206 | 1.5120 |  |  |  |  |
| hsa-miR-551a | 1.4887 |  |  |  |  |
| hsa-miR-486-5p | 1.4820 |  |  |  |  |
| hsa-miR-378a-3p | 1.4820 |  |  |  |  |
| hsa-miR-511 | -1.4813 | * |  |  |  |
| hsa-miR-647 | -1.4713 | * |  |  |  |
| hsa-miR-490-5p | -1.4613 | * |  |  |  |
| hsa-miR-337-3p | -1.4580 |  |  |  |  |
| hsa-miR-1296 | -1.4313 | * |  |  |  |
| hsa-miR-877-3p | -1.4313 |  |  |  |  |
| hsa-miR-183-5p | 1.4220 |  |  |  |  |
| hsa-miR-339-3p | -1.4213 |  |  |  |  |
| hsa-miR-181c-3p | 1.4187 |  |  |  |  |
| hsa-miR-30e-3p | -1.4080 |  |  |  |  |
| hsa-miR-1 | -1.4080 |  |  |  |  |
| hsa-miR-144-5p | 1.3887 |  |  |  |  |
| hsa-miR-330-5p | 1.3387 | * |  |  |  |
| hsa-miR-888-3p | 1.3387 | * |  |  |  |
| hsa-miR-1538 | 1.3387 | * |  |  |  |
| hsa-miR-770-5p | 1.3387 | * |  |  |  |
| hsa-miR-1183 | 1.3387 | * |  |  |  |
| hsa-miR-100-3p | 1.3387 | * |  |  |  |
| hsa-miR-937-3p | 1.3387 | * |  |  |  |
| hsa-miR-499a-3p | 1.3387 | * |  |  |  |
| hsa-miR-621 | 1.3387 | * |  |  |  |
| hsa-miR-601 | 1.3387 | * |  |  |  |
| hsa-miR-517-5p | 1.3387 | * |  |  |  |
| hsa-miR-619 | 1.3387 | * |  |  |  |
| hsa-miR-1237-3p | 1.3387 | * |  |  |  |
| hsa-miR-194-3p | 1.3387 | * |  |  |  |
| hsa-miR-455-3p | 1.3387 | * |  |  |  |
| hsa-miR-135a-3p | 1.3387 | * |  |  |  |
| hsa-miR-1238-3p | 1.3387 | * |  |  |  |
| hsa-miR-615-5p | 1.3387 | * |  |  |  |
| hsa-miR-302e | 1.3387 | * |  |  |  |
| hsa-miR-1914-5p | 1.3387 | * |  |  |  |
| hsa-miR-1181 | 1.3387 | * |  |  |  |
| hsa-miR-520g | 1.3387 | * |  |  |  |
| hsa-miR-577 | 1.3387 | * |  |  |  |
| hsa-miR-432-3p | 1.3387 | * |  |  |  |
| hsa-miR-191-3p | 1.3387 | * |  |  |  |
| hsa-miR-920 | 1.3387 | * |  |  |  |
| hsa-miR-302d-5p | 1.3387 | * |  |  |  |
| hsa-miR-376a-5p | 1.3387 | * |  |  |  |
| hsa-miR-106a-3p | 1.3387 | * |  |  |  |
| hsa-miR-580 | 1.3387 | * |  |  |  |
| hsa-miR-1205 | 1.3387 | * |  |  |  |
| hsa-miR-935 | 1.3387 | * |  |  |  |
| hsa-miR-182-3p | 1.3387 | * |  |  |  |
| hsa-miR-573 | 1.3387 | * |  |  |  |
| hsa-miR-19b-1-5p | 1.3387 | * |  |  |  |
| hsa-miR-610 | 1.3387 | * |  |  |  |
| hsa-miR-1468 | 1.3387 | * |  |  |  |
| hsa-miR-188-3p | 1.3387 | * |  |  |  |
| hsa-miR-515-5p | 1.3387 | * |  |  |  |
| hsa-miR-34a-3p | 1.3387 | * |  |  |  |
| hsa-miR-520d-3p | 1.3387 | * |  |  |  |
| hsa-miR-518f-5p | 1.3387 | * |  |  |  |
| hsa-miR-548d-3p | 1.3387 | * |  |  |  |
| hsa-miR-1911-3p | 1.3387 | * |  |  |  |
| hsa-miR-92a-2-5p | 1.3387 | * |  |  |  |
| hsa-miR-27b-5p | 1.3387 | * |  |  |  |
| hsa-miR-541-3p | 1.3387 | * |  |  |  |
| hsa-miR-519e-3p | 1.3387 | * |  |  |  |
| hsa-miR-488-5p | 1.3387 | * |  |  |  |
| hsa-miR-92a-1-5p | 1.3387 | * |  |  |  |
| hsa-miR-219-1-3p | 1.3387 | * |  |  |  |
| hsa-miR-522-3p | 1.3387 | * |  |  |  |
| hsa-miR-323a-5p | 1.3387 | * |  |  |  |
| hsa-miR-922 | 1.3387 | * |  |  |  |
| hsa-miR-124-5p | 1.3387 | * |  |  |  |
| hsa-miR-1264 | 1.3387 | * |  |  |  |
| hsa-miR-504 | 1.3387 | * |  |  |  |
| hsa-miR-138-1-3p | 1.3387 | * |  |  |  |
| hsa-miR-567 | 1.3387 | * |  |  |  |
| hsa-miR-18b-3p | 1.3387 | * |  |  |  |
| hsa-miR-125a-3p | 1.3387 | * |  |  |  |
| hsa-miR-653 | 1.3387 | * |  |  |  |
| hsa-miR-891b | 1.3387 | * |  |  |  |
| hsa-miR-384 | 1.3387 | * |  |  |  |
| hsa-miR-649 | 1.3387 | * |  |  |  |
| hsa-miR-943 | 1.3387 | * |  |  |  |
| hsa-miR-200b-5p | 1.3387 | * |  |  |  |
| hsa-miR-519e-5p | 1.3387 | * |  |  |  |
| hsa-miR-450b-3p | 1.3387 | * |  |  |  |
| hsa-miR-553 | 1.3387 | * |  |  |  |
| hsa-miR-759 | 1.3387 | * |  |  |  |
| hsa-miR-507 | 1.3387 | * |  |  |  |
| hsa-miR-520b | 1.3387 | * |  |  |  |
| hsa-miR-302f | 1.3387 | * |  |  |  |
| hsa-miR-875-5p | 1.3387 | * |  |  |  |
| hsa-miR-219-2-3p | 1.3387 | * |  |  |  |
| hsa-miR-1244 | 1.3387 | * |  |  |  |
| hsa-miR-566 | 1.3387 | * |  |  |  |
| hsa-miR-1256 | 1.3387 | * |  |  |  |
| hsa-miR-516a-3p | 1.3387 | * |  |  |  |
| hsa-miR-876-3p | 1.3387 | * |  |  |  |
| hsa-miR-659-3p | 1.3387 | * |  |  |  |
| hsa-miR-135b-3p | 1.3387 | * |  |  |  |
| hsa-miR-2113 | 1.3387 | * |  |  |  |
| hsa-miR-1254 | 1.3387 | * |  |  |  |
| hsa-miR-661 | 1.3387 | * |  |  |  |
| hsa-miR-892a | 1.3387 | * |  |  |  |
| hsa-miR-769-3p | 1.3387 | * |  |  |  |
| hsa-miR-518e-5p | 1.3387 | * |  |  |  |
| hsa-miR-489 | 1.3387 | * |  |  |  |
| hsa-miR-381-5p | 1.3387 | * |  |  |  |
| hsa-miR-609 | 1.3387 | * |  |  |  |
| hsa-miR-10a-3p | 1.3387 | * |  |  |  |
| hsa-let-7e-3p | 1.3387 | * |  |  |  |
| hsa-miR-513b | 1.3387 | * |  |  |  |
| hsa-miR-1914-3p | 1.3387 | * |  |  |  |
| hsa-miR-323b-5p | 1.3387 | * |  |  |  |
| hsa-miR-548i | 1.3387 | * |  |  |  |
| hsa-miR-1272 | 1.3387 | * |  |  |  |
| hsa-miR-544a | 1.3387 | * |  |  |  |
| hsa-miR-431-3p | 1.3387 | * |  |  |  |
| hsa-miR-556-5p | 1.3387 | * |  |  |  |
| hsa-miR-1267 | 1.3387 | * |  |  |  |
| hsa-miR-141-5p | 1.3387 | * |  |  |  |
| hsa-miR-222-5p | 1.3387 | * |  |  |  |
| hsa-miR-924 | 1.3387 | * |  |  |  |
| hsa-let-7a-2-3p | 1.3387 | * |  |  |  |
| hsa-miR-520f | 1.3387 | * |  |  |  |
| hsa-miR-520a-3p | 1.3387 | * |  |  |  |
| hsa-miR-548m | 1.3387 | * |  |  |  |
| hsa-miR-448 | 1.3387 | * |  |  |  |
| hsa-miR-19b-2-5p | 1.3387 | * |  |  |  |
| hsa-miR-558 | 1.3387 | * |  |  |  |
| hsa-miR-1258 | 1.3387 | * |  |  |  |
| hsa-miR-624-3p | 1.3387 | * |  |  |  |
| hsa-miR-767-5p | 1.3387 | * |  |  |  |
| hsa-miR-559 | 1.3387 | * |  |  |  |
| hsa-miR-449b-3p | 1.3387 | * |  |  |  |
| hsa-miR-205-3p | 1.3387 | * |  |  |  |
| hsa-miR-149-3p | 1.3387 | * |  |  |  |
| hsa-miR-612 | 1.3387 | * |  |  |  |
| hsa-miR-365b-5p | 1.3387 | * |  |  |  |
| hsa-miR-508-5p | 1.3387 | * |  |  |  |
| hsa-miR-591 | 1.3387 | * |  |  |  |
| hsa-miR-519b-3p | 1.3387 | * |  |  |  |
| hsa-miR-518d-5p | 1.3387 | * |  |  |  |
| hsa-miR-212-5p | 1.3387 | * |  |  |  |
| hsa-miR-520e | 1.3387 | * |  |  |  |
| hsa-miR-646 | 1.3387 | * |  |  |  |
| hsa-miR-626 | 1.3387 | * |  |  |  |
| hsa-miR-586 | 1.3387 | * |  |  |  |
| hsa-miR-103b | 1.3387 | * |  |  |  |
| hsa-miR-192-3p | 1.3387 | * |  |  |  |
| hsa-miR-1909-3p | 1.3387 | * |  |  |  |
| hsa-miR-302b-5p | 1.3387 | * |  |  |  |
| hsa-miR-551b-5p | 1.3387 | * |  |  |  |
| hsa-miR-635 | 1.3387 | * |  |  |  |
| hsa-miR-518d-3p | 1.3387 | * |  |  |  |
| hsa-miR-569 | 1.3387 | * |  |  |  |
| hsa-miR-218-2-3p | 1.3387 | * |  |  |  |
| hsa-miR-519c-3p | 1.3387 | * |  |  |  |
| hsa-miR-554 | 1.3387 | * |  |  |  |
| hsa-miR-938 | 1.3387 | * |  |  |  |
| hsa-miR-1243 | 1.3387 | * |  |  |  |
| hsa-miR-512-3p | 1.3387 | * |  |  |  |
| hsa-miR-587 | 1.3387 | * |  |  |  |
| hsa-miR-603 | 1.3387 | * |  |  |  |
| hsa-miR-1184 | 1.3387 | * |  |  |  |
| hsa-miR-588 | 1.3387 | * |  |  |  |
| hsa-miR-582-3p | 1.3387 | * |  |  |  |
| hsa-miR-452-3p | 1.3387 | * |  |  |  |
| hsa-miR-200c-5p | 1.3387 | * |  |  |  |
| hsa-miR-520a-5p | 1.3387 | * |  |  |  |
| hsa-miR-195-3p | 1.3387 | * |  |  |  |
| hsa-miR-578 | 1.3387 | * |  |  |  |
| hsa-miR-875-3p | 1.3387 | * |  |  |  |
| hsa-miR-876-5p | 1.3387 | * |  |  |  |
| hsa-miR-138-2-3p | 1.3387 | * |  |  |  |
| hsa-miR-581 | 1.3387 | * |  |  |  |
| hsa-miR-1204 | 1.3387 | * |  |  |  |
| hsa-miR-555 | 1.3387 | * |  |  |  |
| hsa-miR-1224-3p | 1.3387 | * |  |  |  |
| hsa-miR-1539 | 1.3387 | * |  |  |  |
| hsa-miR-663b | 1.3387 | * |  |  |  |
| hsa-miR-634 | 1.3387 | * |  |  |  |
| hsa-miR-1248 | 1.3387 | * |  |  |  |
| hsa-miR-1227-3p | 1.3387 | * |  |  |  |
| hsa-miR-513a-3p | 1.3387 | * |  |  |  |
| hsa-miR-645 | 1.3387 | * |  |  |  |
| hsa-miR-34b-5p | 1.3387 | * |  |  |  |
| hsa-miR-614 | 1.3387 | * |  |  |  |
| hsa-miR-616-5p | 1.3387 | * |  |  |  |
| hsa-miR-616-3p | 1.3387 | * |  |  |  |
| hsa-miR-606 | 1.3387 | * |  |  |  |
| hsa-miR-367-5p | 1.3387 | * |  |  |  |
| hsa-miR-1265 | 1.3387 | * |  |  |  |
| hsa-miR-548a-5p | 1.3387 | * |  |  |  |
| hsa-miR-1253 | 1.3387 | * |  |  |  |
| hsa-miR-607 | 1.3387 | * |  |  |  |
| hsa-miR-1208 | 1.3387 | * |  |  |  |
| hsa-miR-1206 | 1.3387 | * |  |  |  |
| hsa-miR-1270 | 1.3387 | * |  |  |  |
| hsa-miR-525-3p | 1.3387 | * |  |  |  |
| hsa-miR-1200 | 1.3387 | * |  |  |  |
| hsa-miR-1911-5p | 1.3387 | * |  |  |  |
| hsa-miR-33b-3p | 1.3387 | * |  |  |  |
| hsa-miR-380-5p | 1.3387 | * |  |  |  |
| hsa-miR-92b-5p | 1.3387 | * |  |  |  |
| hsa-miR-218-1-3p | 1.3387 | * |  |  |  |
| hsa-miR-593-5p | 1.3387 | * |  |  |  |
| hsa-miR-561-3p | 1.3387 | * |  |  |  |
| hsa-miR-767-3p | 1.3387 | * |  |  |  |
| hsa-miR-526b-3p | 1.3387 | * |  |  |  |
| hsa-miR-193b-5p | 1.3387 | * |  |  |  |
| hsa-miR-541-5p | 1.3387 | * |  |  |  |
| hsa-miR-630 | 1.3387 | * |  |  |  |
| hsa-miR-2053 | 1.3387 | * |  |  |  |
| hsa-miR-675-5p | 1.3387 | * |  |  |  |
| hsa-miR-1252 | 1.3387 | * |  |  |  |
| hsa-miR-513c-5p | 1.3387 | * |  |  |  |
| hsa-miR-1182 | 1.3387 | * |  |  |  |
| hsa-miR-611 | 1.3387 | * |  |  |  |
| hsa-miR-515-3p | 1.3387 | * |  |  |  |
| hsa-miR-650 | 1.3387 | * |  |  |  |
| hsa-miR-1178-3p | 1.3387 | * |  |  |  |
| hsa-miR-600 | 1.3387 | * |  |  |  |
| hsa-miR-599 | 1.3387 | * |  |  |  |
| hsa-miR-564 | 1.3387 | * |  |  |  |
| hsa-miR-132-5p | 1.3387 | * |  |  |  |
| hsa-miR-155-3p | 1.3387 | * |  |  |  |
| hsa-miR-7-2-3p | 1.3387 | * |  |  |  |
| hsa-miR-593-3p | 1.3387 | * |  |  |  |
| hsa-miR-1912 | 1.3387 | * |  |  |  |
| hsa-miR-936 | 1.3387 | * |  |  |  |
| hsa-miR-183-3p | 1.3387 | * |  |  |  |
| hsa-miR-562 | 1.3387 | * |  |  |  |
| hsa-miR-130a-5p | 1.3387 | * |  |  |  |
| hsa-miR-563 | 1.3387 | * |  |  |  |
| hsa-miR-200a-5p | 1.3387 | * |  |  |  |
| hsa-miR-15a-3p | 1.3387 | * |  |  |  |
| hsa-miR-944 | 1.3387 | * |  |  |  |
| hsa-miR-548n | 1.3387 | * |  |  |  |
| hsa-miR-638 | 1.3387 | * |  |  |  |
| hsa-miR-10b-3p | 1.3387 | * |  |  |  |
| hsa-miR-571 | 1.3387 | * |  |  |  |
| hsa-miR-1207-5p | 1.3387 | * |  |  |  |
| hsa-miR-300 | 1.3387 | * |  |  |  |
| hsa-miR-1269a | 1.3387 | * |  |  |  |
| hsa-miR-208a | 1.3387 | * |  |  |  |
| hsa-miR-552 | 1.3387 | * |  |  |  |
| hsa-miR-639 | 1.3387 | * |  |  |  |
| hsa-miR-636 | 1.3387 | * |  |  |  |
| hsa-miR-146a-3p | 1.3387 | * |  |  |  |
| hsa-miR-548e | 1.3387 | * |  |  |  |
| hsa-miR-296-3p | 1.3387 | * |  |  |  |
| hsa-miR-663a | 1.3220 |  |  |  |  |
| hsa-miR-16-2-3p | 1.3187 |  |  |  |  |
| hsa-miR-144-3p | 1.3020 | * |  |  |  |
| hsa-miR-502-5p | 1.3020 |  |  |  |  |
| hsa-miR-496 | -1.2813 |  |  |  |  |
| hsa-miR-199a-5p | -1.2680 |  |  |  |  |
| hsa-miR-629-3p | 1.2587 |  |  |  |  |
| hsa-miR-339-5p | -1.2580 |  |  |  |  |
| hsa-miR-885-3p | 1.2487 |  |  |  |  |
| hsa-miR-624-5p | 1.2487 |  |  |  |  |
| hsa-miR-15a-5p | 1.2320 |  |  |  |  |
| hsa-miR-362-5p | 1.2320 |  |  |  |  |
| hsa-miR-548c-5p | 1.2287 |  |  |  |  |
| hsa-miR-133a | -1.2180 |  |  |  |  |
| hsa-miR-505-5p | 1.2087 |  |  |  |  |
| hsa-miR-379-5p | -1.2080 |  |  |  |  |
| hsa-miR-491-5p | -1.2080 |  |  |  |  |
| hsa-miR-29a-3p | 1.2020 |  |  |  |  |
| hsa-miR-99b-3p | -1.2013 | * |  |  |  |
| hsa-miR-139-3p | 1.1987 |  |  |  |  |
| hsa-miR-1471 | 1.1787 |  |  |  |  |
| hsa-miR-21-3p | -1.1680 |  |  |  |  |
| hsa-miR-485-3p | 1.1520 |  |  |  |  |
| hsa-miR-340-5p | -1.1480 |  |  |  |  |
| hsa-miR-150-5p | 1.1420 |  |  |  |  |
| hsa-miR-331-3p | -1.1380 |  |  |  |  |
| hsa-miR-26b-3p | 1.1287 |  |  |  |  |
| hsa-miR-375 | 1.1220 |  |  |  |  |
| hsa-miR-374b-3p | 1.1120 | * |  |  |  |
| hsa-miR-765 | 1.1120 | * |  |  |  |
| hsa-miR-518c-5p | 1.1120 | * |  |  |  |
| hsa-miR-491-3p | 1.1120 | * |  |  |  |
| hsa-miR-30c-2-3p | 1.1120 | * |  |  |  |
| hsa-miR-509-3-5p | 1.1120 | * |  |  |  |
| hsa-miR-138-5p | 1.1120 | * |  |  |  |
| hsa-miR-887 | 1.1120 | * |  |  |  |
| hsa-miR-30b-3p | 1.1120 | * |  |  |  |
| hsa-miR-422a | 1.1120 | * |  |  |  |
| hsa-miR-608 | 1.1120 | * |  |  |  |
| hsa-miR-512-5p | 1.1120 | * |  |  |  |
| hsa-miR-96-3p | 1.1120 | * |  |  |  |
| hsa-miR-185-3p | 1.1120 | * |  |  |  |
| hsa-miR-597 | 1.1120 | * |  |  |  |
| hsa-miR-373-5p | 1.1120 | * |  |  |  |
| hsa-miR-149-5p | 1.1120 | * |  |  |  |
| hsa-miR-455-5p | 1.1120 | * |  |  |  |
| hsa-miR-299-3p | 1.1120 | * |  |  |  |
| hsa-miR-154-3p | 1.1120 | * |  |  |  |
| hsa-miR-218-5p | 1.1120 | * |  |  |  |
| hsa-miR-34c-5p | 1.1120 | * |  |  |  |
| hsa-miR-217 | 1.1120 | * |  |  |  |
| hsa-miR-623 | 1.1120 | * |  |  |  |
| hsa-miR-520c-3p | 1.1120 | * |  |  |  |
| hsa-miR-557 | 1.1120 | * |  |  |  |
| hsa-miR-31-3p | 1.1120 | * |  |  |  |
| hsa-miR-325 | 1.1120 | * |  |  |  |
| hsa-miR-137 | 1.1120 | * |  |  |  |
| hsa-miR-524-3p | 1.1120 | * |  |  |  |
| hsa-miR-890 | 1.1120 | * |  |  |  |
| hsa-miR-524-5p | 1.1120 | * |  |  |  |
| hsa-miR-933 | 1.1120 | * |  |  |  |
| hsa-miR-668 | 1.1120 | * |  |  |  |
| hsa-miR-888-5p | 1.1120 | * |  |  |  |
| hsa-miR-518c-3p | 1.1120 | * |  |  |  |
| hsa-miR-367-3p | 1.1120 | * |  |  |  |
| hsa-miR-302c-3p | 1.1120 | * |  |  |  |
| hsa-miR-129-5p | 1.1120 | * |  |  |  |
| hsa-miR-492 | 1.1120 | * |  |  |  |
| hsa-miR-302d-3p | 1.1120 | * |  |  |  |
| hsa-miR-202-3p | 1.1120 | * |  |  |  |
| hsa-miR-506-3p | 1.1120 | * |  |  |  |
| hsa-miR-516b-5p | 1.1120 | * |  |  |  |
| hsa-miR-548b-3p | 1.1120 | * |  |  |  |
| hsa-miR-302b-3p | 1.1120 | * |  |  |  |
| hsa-miR-662 | 1.1120 | * |  |  |  |
| hsa-miR-519d | 1.1120 | * |  |  |  |
| hsa-miR-371a-3p | 1.1120 | * |  |  |  |
| hsa-miR-637 | 1.1120 | * |  |  |  |
| hsa-miR-631 | 1.1120 | * |  |  |  |
| hsa-miR-518e-3p | 1.1120 | * |  |  |  |
| hsa-miR-658 | 1.1120 | * |  |  |  |
| hsa-miR-572 | 1.1120 | * |  |  |  |
| hsa-miR-802 | 1.1120 | * |  |  |  |
| hsa-miR-521 | 1.1120 | * |  |  |  |
| hsa-miR-526b-5p | 1.1120 | * |  |  |  |
| hsa-miR-184 | 1.1120 | * |  |  |  |
| hsa-miR-519a-3p | 1.1120 | * |  |  |  |
| hsa-miR-129-2-3p | 1.1120 | * |  |  |  |
| hsa-miR-488-3p | 1.1120 | * |  |  |  |
| hsa-miR-371a-5p | 1.1120 | * |  |  |  |
| hsa-miR-891a | 1.1120 | * |  |  |  |
| hsa-miR-549a | 1.1120 | * |  |  |  |
| hsa-miR-518b | 1.1120 | * |  |  |  |
| hsa-miR-202-5p | 1.1120 | * |  |  |  |
| hsa-miR-548c-3p | 1.1120 | * |  |  |  |
| hsa-miR-372 | 1.1120 | * |  |  |  |
| hsa-miR-302a-3p | 1.1120 | * |  |  |  |
| hsa-miR-595 | 1.1120 | * |  |  |  |
| hsa-miR-602 | 1.1120 | * |  |  |  |
| hsa-miR-514a-3p | 1.1120 | * |  |  |  |
| hsa-miR-498 | 1.1120 | * |  |  |  |
| hsa-miR-135b-5p | 1.1120 | * |  |  |  |
| hsa-miR-596 | 1.1120 | * |  |  |  |
| hsa-miR-622 | 1.1120 | * |  |  |  |
| hsa-miR-516a-5p | 1.1120 | * |  |  |  |
| hsa-miR-615-3p | 1.1120 | * |  |  |  |
| hsa-miR-298 | 1.1120 | * |  |  |  |
| hsa-miR-449b-5p | 1.1120 | * |  |  |  |
| hsa-miR-520d-5p | 1.1120 | * |  |  |  |
| hsa-miR-383 | 1.1120 | * |  |  |  |
| hsa-miR-363-5p | 1.1120 | * |  |  |  |
| hsa-miR-147b | 1.1120 | * |  |  |  |
| hsa-miR-105-5p | 1.1120 | * |  |  |  |
| hsa-miR-147a | 1.1120 | * |  |  |  |
| hsa-miR-198 | 1.1120 | * |  |  |  |
| hsa-miR-517a-3p | 1.1120 | * |  |  |  |
| hsa-miR-518f-3p | 1.1120 | * |  |  |  |
| hsa-miR-617 | 1.1120 | * |  |  |  |
| hsa-miR-708-5p | 1.1120 | * |  |  |  |
| hsa-miR-517c-3p | 1.1120 | * |  |  |  |
| hsa-miR-412 | 1.1120 | * |  |  |  |
| hsa-miR-216a-5p | 1.1120 | * |  |  |  |
| hsa-miR-921 | 1.1120 | * |  |  |  |
| hsa-miR-513a-5p | 1.1120 | * |  |  |  |
| hsa-miR-216b | 1.1120 | * |  |  |  |
| hsa-miR-510 | 1.1120 | * |  |  |  |
| hsa-miR-525-5p | 1.1120 | * |  |  |  |
| hsa-miR-583 | 1.1120 | * |  |  |  |
| hsa-miR-429 | 1.1120 | * |  |  |  |
| hsa-miR-518a-3p | 1.1120 | * |  |  |  |
| hsa-miR-508-3p | 1.1120 | * |  |  |  |
| hsa-miR-620 | 1.1120 | * |  |  |  |
| hsa-miR-523-3p | 1.1120 | * |  |  |  |
| hsa-miR-20b-3p | 1.1120 | * |  |  |  |
| hsa-miR-550a-5p | 1.1120 | * |  |  |  |
| hsa-miR-302c-5p | 1.1120 | * |  |  |  |
| hsa-miR-34c-3p | 1.1120 | * |  |  |  |
| hsa-miR-576-5p | 1.1120 | * |  |  |  |
| hsa-miR-542-5p | -1.1080 |  |  |  |  |
| hsa-miR-744-3p | -1.0813 | * |  |  |  |
| hsa-miR-369-3p | -1.0813 |  |  |  |  |
| hsa-miR-15b-3p | 1.0587 |  |  |  |  |
| hsa-miR-651 | 1.0520 |  |  |  |  |
| hsa-miR-133b | -1.0380 |  |  |  |  |
| hsa-miR-99b-5p | -0.9980 |  |  |  |  |
| hsa-miR-433 | -0.9880 |  |  |  |  |
| hsa-miR-326 | -0.9880 |  |  |  |  |
| hsa-miR-744-5p | -0.9680 |  |  |  |  |
| hsa-miR-106b-3p | -0.9613 |  |  |  |  |
| hsa-miR-143-5p | -0.9613 | * |  |  |  |
| hsa-miR-1260a | 0.9587 |  |  |  |  |
| hsa-miR-503-5p | -0.9480 |  |  |  |  |
| hsa-miR-451a | 0.9420 |  |  |  |  |
| hsa-miR-660-5p | 0.9420 |  |  |  |  |
| hsa-miR-148b-5p | -0.9413 | * |  |  |  |
| hsa-miR-590-3p | 0.9387 |  |  |  |  |
| hsa-miR-532-5p | 0.9320 |  |  |  |  |
| hsa-miR-766-3p | -0.9280 |  |  |  |  |
| hsa-miR-758-3p | 0.9187 | * |  |  |  |
| hsa-miR-337-5p | -0.9180 |  |  |  |  |
| hsa-miR-411-3p | 0.8987 | * |  |  |  |
| hsa-miR-130b-3p | -0.8880 |  |  |  |  |
| hsa-miR-27a-3p | -0.8880 |  |  |  |  |
| hsa-let-7e-5p | -0.8780 |  |  |  |  |
| hsa-miR-195-5p | -0.8680 |  |  |  |  |
| hsa-miR-16-5p | 0.8620 |  |  |  |  |
| hsa-miR-127-3p | -0.8580 |  |  |  |  |
| hsa-miR-211-5p | -0.8480 | * |  |  |  |
| hsa-miR-382-5p | -0.8480 |  |  |  |  |
| hsa-miR-27b-3p | -0.8480 |  |  |  |  |
| hsa-miR-409-3p | -0.8280 |  |  |  |  |
| hsa-miR-335-5p | -0.8280 |  |  |  |  |
| hsa-miR-10b-5p | 0.8220 |  |  |  |  |
| hsa-miR-222-3p | 0.8220 |  |  |  |  |
| hsa-miR-151a-3p | -0.8080 |  |  |  |  |
| hsa-let-7f-2-3p | -0.8013 |  |  |  |  |
| hsa-miR-125a-5p | -0.7980 |  |  |  |  |
| hsa-miR-22-5p | 0.7820 |  |  |  |  |
| hsa-miR-214-3p | -0.7680 | * |  |  |  |
| hsa-miR-132-3p | 0.7520 |  |  |  |  |
| hsa-miR-424-3p | 0.7487 |  |  |  |  |
| hsa-miR-548l | 0.7387 | * |  |  |  |
| hsa-miR-885-5p | -0.7180 |  |  |  |  |
| hsa-miR-450a-5p | 0.7120 | * |  |  |  |
| hsa-miR-208b | -0.7113 | * |  |  |  |
| hsa-miR-335-3p | -0.7113 |  |  |  |  |
| hsa-miR-490-3p | 0.7020 |  |  |  |  |
| hsa-miR-145-3p | 0.6987 |  |  |  |  |
| hsa-miR-143-3p | -0.6980 |  |  |  |  |
| hsa-miR-18a-5p | -0.6780 |  |  |  |  |
| hsa-miR-346 | 0.6720 |  |  |  |  |
| hsa-miR-10a-5p | 0.6720 |  |  |  |  |
| hsa-miR-889 | -0.6713 |  |  |  |  |
| hsa-miR-421 | -0.6680 |  |  |  |  |
| hsa-miR-548a-3p | -0.6613 |  |  |  |  |
| hsa-miR-187-5p | -0.6480 | * |  |  |  |
| hsa-miR-584-5p | -0.6480 |  |  |  |  |
| hsa-miR-25-3p | 0.6420 |  |  |  |  |
| hsa-miR-32-5p | 0.6420 |  |  |  |  |
| hsa-miR-671-3p | -0.6413 |  |  |  |  |
| hsa-miR-664a-3p | 0.6387 |  |  |  |  |
| hsa-miR-655 | 0.6387 |  |  |  |  |
| hsa-miR-92a-3p | 0.6320 |  |  |  |  |
| hsa-miR-296-5p | 0.6320 |  |  |  |  |
| hsa-miR-625-3p | 0.6220 |  |  |  |  |
| hsa-miR-494 | -0.6180 |  |  |  |  |
| hsa-miR-338-5p | 0.6087 |  |  |  |  |
| hsa-miR-26a-2-3p | 0.6020 | * |  |  |  |
| hsa-miR-215 | 0.6020 |  |  |  |  |
| hsa-miR-1247-5p | -0.6013 | * |  |  |  |
| hsa-miR-33a-5p | -0.5980 |  |  |  |  |
| hsa-miR-940 | 0.5920 |  |  |  |  |
| hsa-miR-152 | -0.5880 |  |  |  |  |
| hsa-miR-450b-5p | 0.5787 |  |  |  |  |
| hsa-miR-7-1-3p | 0.5787 |  |  |  |  |
| hsa-miR-221-3p | -0.5780 |  |  |  |  |
| hsa-miR-141-3p | 0.5720 |  |  |  |  |
| hsa-miR-486-3p | 0.5587 |  |  |  |  |
| hsa-miR-320d | 0.5587 |  |  |  |  |
| hsa-miR-33b-5p | -0.5580 |  |  |  |  |
| hsa-let-7b-5p | 0.5520 |  |  |  |  |
| hsa-miR-125b-5p | 0.5520 |  |  |  |  |
| hsa-miR-532-3p | -0.5513 |  |  |  |  |
| hsa-miR-361-5p | -0.5480 |  |  |  |  |
| hsa-miR-548j | -0.5413 |  |  |  |  |
| hsa-miR-34a-5p | -0.5280 |  |  |  |  |
| hsa-miR-320c | 0.5087 |  |  |  |  |
| hsa-miR-145-5p | -0.5080 |  |  |  |  |
| hsa-miR-30b-5p | -0.4980 |  |  |  |  |
| hsa-miR-194-5p | 0.4920 |  |  |  |  |
| hsa-miR-628-5p | 0.4887 |  |  |  |  |
| hsa-miR-223-5p | 0.4887 |  |  |  |  |
| hsa-miR-28-5p | -0.4880 |  |  |  |  |
| hsa-miR-942 | 0.4687 |  |  |  |  |
| hsa-miR-640 | -0.4613 | * |  |  |  |
| hsa-miR-146b-5p | -0.4580 |  |  |  |  |
| hsa-miR-197-3p | -0.4580 |  |  |  |  |
| hsa-miR-122-5p | -0.4580 |  |  |  |  |
| hsa-miR-24-3p | -0.4580 |  |  |  |  |
| hsa-miR-148a-3p | 0.4520 |  |  |  |  |
| hsa-miR-124-3p | 0.4420 |  |  |  |  |
| hsa-miR-30c-5p | 0.4420 |  |  |  |  |
| hsa-miR-29b-2-5p | 0.4420 |  |  |  |  |
| hsa-miR-23b-3p | -0.4380 |  |  |  |  |
| hsa-miR-342-3p | 0.4320 |  |  |  |  |
| hsa-let-7b-3p | -0.4313 |  |  |  |  |
| hsa-miR-324-5p | -0.4280 |  |  |  |  |
| hsa-miR-574-3p | -0.4280 |  |  |  |  |
| hsa-miR-146a-5p | -0.4280 |  |  |  |  |
| hsa-miR-18b-5p | -0.4280 |  |  |  |  |
| hsa-miR-99a-5p | -0.4180 |  |  |  |  |
| hsa-miR-140-5p | -0.4180 |  |  |  |  |
| hsa-miR-30a-5p | 0.4120 |  |  |  |  |
| hsa-miR-146b-3p | -0.4113 |  |  |  |  |
| hsa-miR-643 | 0.4087 |  |  |  |  |
| hsa-miR-188-5p | -0.4080 |  |  |  |  |
| hsa-miR-151a-5p | -0.4080 |  |  |  |  |
| hsa-miR-423-3p | 0.4020 |  |  |  |  |
| hsa-miR-497-5p | 0.4020 |  |  |  |  |
| hsa-miR-484 | 0.3920 |  |  |  |  |
| hsa-miR-100-5p | 0.3920 |  |  |  |  |
| hsa-miR-181a-5p | -0.3880 |  |  |  |  |
| hsa-miR-126-5p | -0.3880 |  |  |  |  |
| hsa-miR-505-3p | 0.3820 |  |  |  |  |
| hsa-miR-210 | -0.3680 |  |  |  |  |
| hsa-miR-106b-5p | -0.3680 |  |  |  |  |
| hsa-miR-139-5p | -0.3580 |  |  |  |  |
| hsa-miR-652-3p | -0.3580 |  |  |  |  |
| hsa-miR-101-3p | 0.3520 |  |  |  |  |
| hsa-miR-320a | 0.3420 |  |  |  |  |
| hsa-miR-671-5p | 0.3420 | * |  |  |  |
| hsa-miR-106a-5p | -0.3380 |  |  |  |  |
| hsa-miR-328 | -0.3280 |  |  |  |  |
| hsa-miR-204-5p | -0.3180 |  |  |  |  |
| hsa-miR-26a-5p | -0.3180 |  |  |  |  |
| hsa-let-7d-5p | -0.3080 |  |  |  |  |
| hsa-miR-30e-5p | 0.2920 |  |  |  |  |
| hsa-miR-330-3p | 0.2920 |  |  |  |  |
| hsa-miR-1913 | -0.2913 |  |  |  |  |
| hsa-miR-454-3p | -0.2880 |  |  |  |  |
| hsa-miR-345-5p | 0.2820 |  |  |  |  |
| hsa-miR-223-3p | -0.2780 |  |  |  |  |
| hsa-miR-199a-3p | -0.2780 |  |  |  |  |
| hsa-miR-301a-3p | -0.2780 |  |  |  |  |
| hsa-miR-338-3p | -0.2580 |  |  |  |  |
| hsa-let-7f-5p | -0.2580 |  |  |  |  |
| hsa-miR-627 | 0.2520 |  |  |  |  |
| hsa-miR-761 | 0.2487 | * |  |  |  |
| hsa-miR-193a-3p | 0.2420 | * |  |  |  |
| hsa-miR-361-3p | -0.2380 |  |  |  |  |
| hsa-miR-590-5p | -0.2380 |  |  |  |  |
| hsa-let-7i-5p | 0.2320 |  |  |  |  |
| hsa-miR-769-5p | -0.2313 |  |  |  |  |
| hsa-miR-30d-5p | -0.2280 |  |  |  |  |
| hsa-miR-148b-3p | -0.2280 |  |  |  |  |
| hsa-miR-130a-3p | -0.2280 |  |  |  |  |
| hsa-miR-93-3p | -0.2213 |  |  |  |  |
| hsa-miR-30d-3p | 0.2187 |  |  |  |  |
| hsa-miR-22-3p | 0.2120 |  |  |  |  |
| hsa-miR-21-5p | 0.2120 |  |  |  |  |
| hsa-miR-2110 | -0.2113 |  |  |  |  |
| hsa-miR-320b | 0.1987 |  |  |  |  |
| hsa-miR-877-5p | 0.1920 |  |  |  |  |
| hsa-miR-125b-1-3p | -0.1913 | * |  |  |  |
| hsa-miR-1908 | 0.1887 |  |  |  |  |
| hsa-miR-628-3p | -0.1880 |  |  |  |  |
| hsa-miR-642a-5p | -0.1880 |  |  |  |  |
| hsa-miR-17-5p | -0.1780 |  |  |  |  |
| hsa-miR-15b-5p | -0.1780 |  |  |  |  |
| hsa-miR-501-3p | 0.1687 |  |  |  |  |
| hsa-miR-193b-3p | -0.1680 |  |  |  |  |
| hsa-miR-181b-5p | -0.1680 |  |  |  |  |
| hsa-miR-23a-3p | -0.1680 |  |  |  |  |
| hsa-miR-874 | -0.1580 |  |  |  |  |
| hsa-miR-186-5p | 0.1520 |  |  |  |  |
| hsa-miR-107 | 0.1520 |  |  |  |  |
| hsa-miR-98-5p | 0.1520 |  |  |  |  |
| hsa-miR-374a-5p | -0.1480 |  |  |  |  |
| hsa-miR-185-5p | -0.1480 |  |  |  |  |
| hsa-miR-29b-3p | 0.1420 |  |  |  |  |
| hsa-miR-23a-5p | -0.1313 | * |  |  |  |
| hsa-miR-33a-3p | 0.1287 | * |  |  |  |
| hsa-miR-550a-3p | 0.1287 |  |  |  |  |
| hsa-miR-329 | -0.1280 |  |  |  |  |
| hsa-miR-365a-3p | -0.1280 |  |  |  |  |
| hsa-let-7d-3p | -0.1280 |  |  |  |  |
| hsa-miR-17-3p | -0.1213 |  |  |  |  |
| hsa-miR-520h | 0.1087 |  |  |  |  |
| hsa-miR-128 | 0.1020 |  |  |  |  |
| hsa-miR-28-3p | -0.1013 |  |  |  |  |
| hsa-miR-1537 | 0.0987 |  |  |  |  |
| hsa-miR-130b-5p | 0.0987 |  |  |  |  |
| hsa-miR-425-3p | -0.0980 |  |  |  |  |
| hsa-let-7c | 0.0920 |  |  |  |  |
| hsa-miR-224-5p | 0.0920 |  |  |  |  |
| hsa-miR-142-5p | -0.0880 |  |  |  |  |
| hsa-let-7g-5p | -0.0880 |  |  |  |  |
| hsa-miR-324-3p | 0.0820 |  |  |  |  |
| hsa-miR-19b-3p | 0.0720 |  |  |  |  |
| hsa-miR-20b-5p | 0.0520 |  |  |  |  |
| hsa-miR-155-5p | 0.0520 |  |  |  |  |
| hsa-miR-20a-3p | 0.0487 |  |  |  |  |
| hsa-miR-92b-3p | -0.0480 |  |  |  |  |
| hsa-miR-589-5p | -0.0480 | * |  |  |  |
| hsa-miR-199b-5p | 0.0320 |  |  |  |  |
| hsa-miR-192-5p | 0.0320 |  |  |  |  |
| hsa-miR-378a-5p | 0.0287 |  |  |  |  |
| hsa-miR-20a-5p | -0.0280 |  |  |  |  |
| hsa-miR-19a-3p | 0.0220 |  |  |  |  |
| hsa-miR-545-3p | 0.0220 |  |  |  |  |
| hsa-miR-181d | 0.0220 | * |  |  |  |
| hsa-miR-26b-5p | -0.0080 |  |  |  |  |
| hsa-miR-142-3p | 0.0020 |  |  |  |  |
| hsa-miR-126-3p | 0.0020 |  |  |  |  |
| hsa-let-7a-5p | 0.0020 |  |  |  |  |
| hsa-miR-598 | 0.0020 |  |  |  |  |
| *Undifined Cтvalues were substituted by 40. | | |  |  |  |
| Selected miRNAs for further analyses were indicated in red | | | | |  |
