# Supplementary figures and images for: Plasma miRNA expression profiles in rheumatoid arthritis associated interstitial lung disease
Source: BMC Musculoskelet Disord. 2017 Jan 19;18:21. doi: 10.1186/s12891-017-1389-4 (PMC5244611; doi:10.1186/s12891-017-1389-4)

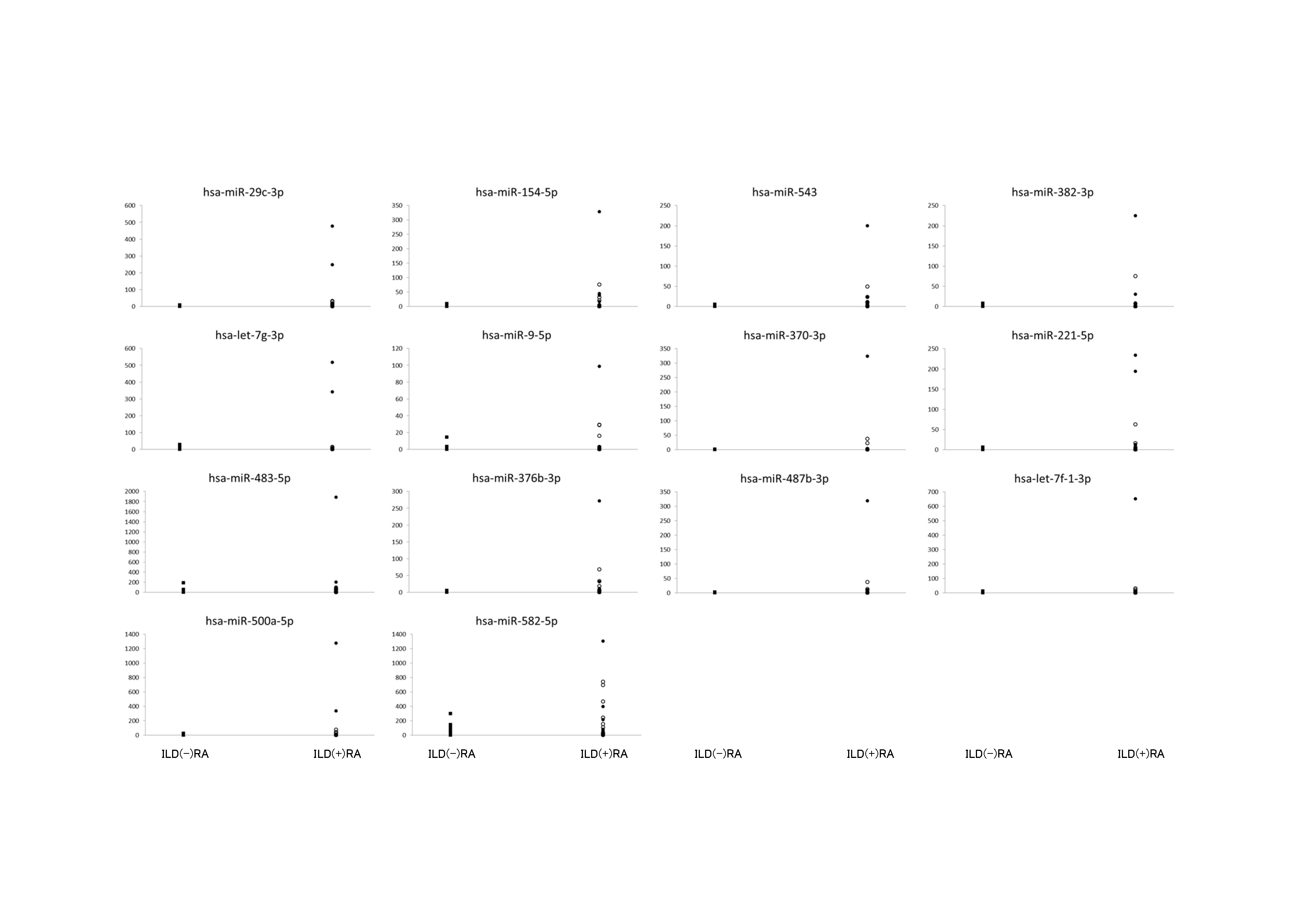

Supplement: Additional file 2: Figure S1. — Distribution of the miRNA, Krebs von den lungen-6 (KL-6), surfactant protein-D (SP-D), ILDIndex levels, as markers for interstitial lung disease (ILD) in rheumatoid arthritis (RA) patients. The filled square, filled circle, and empty circle represent ILD(−)RA, RA with usual interstitial pneumonia, and RA with nonspecific interstitial pneumonia, respectively. ILD(+)RA: RA with ILD, ILD(−)RA: RA without ILD. (TIFF 687 kb) [file 12891_2017_1389_MOESM2_ESM.tiff]
