# Supplementary material for: Plasma miRNA expression profiles in rheumatoid arthritis associated interstitial lung disease
Source: BMC Musculoskelet Disord. 2017 Jan 19;18:21. doi: 10.1186/s12891-017-1389-4 (PMC5244611; doi:10.1186/s12891-017-1389-4)
Supplement: Additional file 3: Table S2. — miRNA profiles of the RA patients with ILD. Standard deviations are shown in parenthesis. Difference were tested between male and female by Mann-Whitney’s U test. RA: rheumatoid arthritis, ILD(+)RA: ILD positive RA. Average values of each group are shown. (DOCX 15 kb) [file 12891_2017_1389_MOESM3_ESM.docx]

| Supplementary Table 2. miRNA profiles of the RA patients with ILD. | | | |
| --- | --- | --- | --- |
|  | ILD(+)RA |  |  |
| miRNA | Male | Female | *P* |
| hsa-miR-29c-3p | 28.5 (72.8) | 28.8 (102.9) | 0.6770 |
| hsa-miR-154-5p | 34.6 (97.8) | 9.2 (19.8) | 0.6248 |
| hsa-miR-543 | 22.2 (59.4) | 5.1 (11.4) | 0.3063 |
| hsa-miR-214-5p | 22.3 (46.1) | 0.7 (1.7) | 0.2266 |
| hsa-miR-382-3p | 21.8 (67.4) | 5.1 (17.2) | 0.3863 |
| hsa-let-7g-3p | 48.9 (155.2) | 18.7 (74.0) | 0.9525 |
| hsa-miR-9-5p | 5.7 (11.5) | 5.8 (21.6) | 0.3816 |
| hsa-miR-370-3p | 29.5 (97.7) | 3.1 (9.2) | 0.4091 |
| hsa-miR-221-5p | 23.4 (69.9) | 13.8 (43.5) | 0.9509 |
| hsa-miR-483-5p | 183.3 (564.9) | 26.9 (47.8) | 0.9842 |
| hsa-miR-7-5p | 9.0 (10.3) | 81.0 (321.2) | 0.8273 |
| hsa-miR-376b-3p | 30.1 (80.6) | 6.5 (15.9) | 0.0689 |
| hsa-miR-487b-3p | 30.9 (95.8) | 3.1 (8.2) | 0.2322 |
| hsa-let-7f-1-3p | 62.0 (195.9) | 5.5 (8.0) | 0.9049 |
| hsa-miR-500a-5p | 38.6 (100.0) | 64.8 (277.9) | 0.2138 |
| hsa-miR-582-5p | 178.6 (279.6) | 127.1 (298.3) | 0.4737 |
| RA: rheumatoid arthritis, ILD(+)RA: ILD positive RA. Average values of each group are shown. Standard deviations are shown in parenthesis. Difference were tested between male and female by Mann-Whitney's U test. | | | |
|  |  |  |  |
|  |  |  |  |
|  |  |  |  |
