# Supplementary material for: Plasma miRNA expression profiles in rheumatoid arthritis associated interstitial lung disease
Source: BMC Musculoskelet Disord. 2017 Jan 19;18:21. doi: 10.1186/s12891-017-1389-4 (PMC5244611; doi:10.1186/s12891-017-1389-4)
Supplement: Additional file 4: Table S3. — miRNA profiles of the RA patients with ILD. Average values of each group are shown. Standard deviations are shown in parenthesis. Difference were tested between age < 70 and age ≧ 70 by Mann-Whitney’s U test. RA: rheumatoid arthritis, ILD(+)RA: ILD positive RA. (DOCX 15 kb) [file 12891_2017_1389_MOESM4_ESM.docx]

| Supplementary Table 3. miRNA profiles of the RA patients with ILD. | | | |
| --- | --- | --- | --- |
|  | ILD(+)RA |  |  |
| miRNA | age <70 | age ≧70 | *P* |
| hsa-miR-29c-3p | 9.1 (10.1) | 46.0 (125.6) | 0.4389 |
| hsa-miR-154-5p | 10.5 (20.3) | 24.4 (79.4) | 0.4378 |
| hsa-miR-543 | 7.7 (13.0) | 13.9 (48.3) | 0.1220 |
| hsa-miR-214-5p | 7.9 (21.0) | 8.3 (33.9) | 0.0560 |
| hsa-miR-382-3p | 6.2 (19.1) | 15.0 (54.5) | 0.4896 |
| hsa-let-7g-3p | 2.8 (4.4) | 52.3 (145.2) | 0.4846 |
| hsa-miR-9-5p | 5.2 (10.5) | 6.2 (23.8) | 0.6320 |
| hsa-miR-370-3p | 4.2 (10.7) | 19.2 (78.6) | 0.1748 |
| hsa-miR-221-5p | 6.2 (16.1) | 26.7 (70.9) | 0.5709 |
| hsa-miR-483-5p | 18.2 (25.8) | 135.8 (453.7) | 0.7482 |
| hsa-miR-7-5p | 13.6 (19.1) | 93.9 (357.5) | 0.4389 |
| hsa-miR-376b-3p | 10.7 (18.0) | 18.1 (65.7) | 0.0260 |
| hsa-miR-487b-3p | 5.0 (9.6) | 19.4 (77.3) | 0.0144 |
| hsa-let-7f-1-3p | 6.5 (8.9) | 41.1 (157.6) | 0.2112 |
| hsa-miR-500a-5p | 10.6 (20.9) | 95.6 (314.8) | 0.6220 |
| hsa-miR-582-5p | 163.2 (258.6) | 128.6 (319.7) | 0.8796 |
| RA: rheumatoid arthritis, ILD(+)RA: ILD positive RA. Average values of each group are shown. Standard deviations are shown in parenthesis. Difference were tested between age < 70 and age ≧ 70 by Mann-Whitney's U test. | | | |
|  |  |  |  |
|  |  |  |  |
|  |  |  |  |
