# Supplementary material for: Plasma miRNA expression profiles in rheumatoid arthritis associated interstitial lung disease
Source: BMC Musculoskelet Disord. 2017 Jan 19;18:21. doi: 10.1186/s12891-017-1389-4 (PMC5244611; doi:10.1186/s12891-017-1389-4)
Supplement: Additional file 5: Table S4. — miRNA profiles of the RA patients with ILD. Average values of each group are shown. Standard deviations are shown in parenthesis. Difference were tested between DAS28 < 4.0 and DAS28 ≧ 4.0 by Mann-Whitney’s U test. RA: rheumatoid arthritis, ILD(+)RA: ILD positive RA. (DOCX 15 kb) [file 12891_2017_1389_MOESM5_ESM.docx]

| Supplementary Table 4. miRNA profiles of the RA patients with ILD. | | | |
| --- | --- | --- | --- |
|  | ILD(+)RA |  |  |
| miRNA | DAS28 <4.0 | DAS28 ≧4.0 | *P* |
| hsa-miR-29c-3p | 25.1 (64.5) | 7.0 (8.4) | 0.3824 |
| hsa-miR-154-5p | 30.9 (87.8) | 7.4 (13.6) | 0.3398 |
| hsa-miR-543 | 19.7 (53.4) | 5.2 (8.5) | 0.7680 |
| hsa-miR-214-5p | 11.6 (37.1) | 7.5 (22.8) | 0.6315 |
| hsa-miR-382-3p | 22.2 (61.6) | 2.8 (8.3) | 0.0500 |
| hsa-let-7g-3p | 39.9 (137.3) | 2.3 (3.3) | 0.4519 |
| hsa-miR-9-5p | 2.6 (7.6) | 3.8 (8.8) | 0.8730 |
| hsa-miR-370-3p | 25.9 (86.4) | 2.1 (6.2) | 0.5086 |
| hsa-miR-221-5p | 23.5 (62.7) | 1.9 (4.3) | 0.2165 |
| hsa-miR-483-5p | 163.7 (498.4) | 14.6 (20.3) | 0.1742 |
| hsa-miR-7-5p | 12.7 (13.2) | 10.8 (18.6) | 0.4375 |
| hsa-miR-376b-3p | 26.4 (72.6) | 7.5 (12.0) | 0.9223 |
| hsa-miR-487b-3p | 26.5 (84.9) | 2.7 (4.3) | 0.4888 |
| hsa-let-7f-1-3p | 51.2 (173.2) | 5.6 (8.7) | 1.0000 |
| hsa-miR-500a-5p | 32.8 (88.9) | 3.7 (7.8) | 0.9797 |
| hsa-miR-582-5p | 128.9 (207.8) | 116.9 (219.8) | 0.7892 |
| RA: rheumatoid arthritis, ILD(+)RA: ILD positive RA. Average values of each group are shown. Standard deviations are shown in parenthesis. Difference were tested between DAS28 < 4.0 and DAS28 ≧　4.0 by Mann-Whitney's U test. | | | |
|  |  |  |  |
|  |  |  |  |
|  |  |  |  |
