# Supplementary material for: Plasma miRNA expression profiles in rheumatoid arthritis associated interstitial lung disease
Source: BMC Musculoskelet Disord. 2017 Jan 19;18:21. doi: 10.1186/s12891-017-1389-4 (PMC5244611; doi:10.1186/s12891-017-1389-4)
Supplement: Additional file 6: Table S5. — miRNA profiles of the RA patients with ILD. Average values of each group are shown. Standard deviations are shown in parenthesis. Difference were tested between bDMARDs(+) and bDMARDs(−) by Mann-Whitney’s U test. RA: rheumatoid arthritis, ILD(+)RA: ILD positive RA. (DOCX 15 kb) [file 12891_2017_1389_MOESM6_ESM.docx]

| Supplementary Table 5. miRNA profiles of the RA patients with ILD. | | | |
| --- | --- | --- | --- |
|  | ILD(+)RA |  |  |
| miRNA | ｂDMARDｓ(-) | ｂDMARDｓ(+) | *P* |
| hsa-miR-29c-3p | 37.8 (110.9) | 8.8 (9.3) | 0.3711 |
| hsa-miR-154-5p | 19.2 (69.7) | 15.0 (25.7) | 0.9500 |
| hsa-miR-543 | 11.4 (42.3) | 10.2 (16.3) | 0.8371 |
| hsa-miR-214-5p | 11.0 (33.8) | 1.9 (3.9) | 0.6911 |
| hsa-miR-382-3p | 10.7 (47.9) | 11.2 (24.1) | 0.1640 |
| hsa-let-7g-3p | 40.3 (128.7) | 4.4 (4.3) | 0.1127 |
| hsa-miR-9-5p | 8.1 (22.1) | 0.6 (1.0) | 0.3131 |
| hsa-miR-370-3p | 15.9 (69.0) | 3.9 (11.6) | 0.8778 |
| hsa-miR-221-5p | 21.2 (62.8) | 8.2 (19.4) | 0.4743 |
| hsa-miR-483-5p | 100.4 (399.5) | 37.3 (61.4) | 0.1933 |
| hsa-miR-7-5p | 77.0 (314.0) | 10.5 (12.2) | 0.4398 |
| hsa-miR-376b-3p | 15.0 (57.4) | 13.8 (23.0) | 0.7276 |
| hsa-miR-487b-3p | 15.6 (67.9) | 6.2 (11.7) | 0.6185 |
| hsa-let-7f-1-3p | 33.2 (138.5) | 6.7 (6.7) | 0.2061 |
| hsa-miR-500a-5p | 79.0 (276.9) | 4.6 (12.4) | 0.1113 |
| hsa-miR-582-5p | 189.8 (339.0) | 45.8 (52.5) | 0.9350 |
| RA: rheumatoid arthritis, ILD(+)RA: ILD positive RA. Average values of each group are shown. Standard deviations are shown in parenthesis. Difference were tested between bDMARDs(+) and bDMARDs(-) by Mann-Whitney's U test. | | | |
|  |  |  |  |
|  |  |  |  |
|  |  |  |  |
